# Leucine-rich repeat kinase 2 biomarkers for Parkinson’s disease

**DOI:** 10.1042/BCJ20253099

**Published:** 2025-05-29

**Authors:** Nicolas Dzamko

**Affiliations:** 1 Faculty of Medicine and Health and the Brain and Mind Centre, University of Sydney, Camperdown, NSW, Australia

**Keywords:** LRRK2, biomarker, lysosome, mitochondria, Rab GTPase

## Abstract

Leucine-rich repeat kinase 2 (LRRK2) has emerged as a promising therapeutic target for the treatment of neurodegenerative Parkinson’s disease (PD). Data from a multitude of pre-clinical models are supportive of a potential role for LRRK2 therapies to ameliorate cellular dysfunctions found in PD, and small molecules to inhibit LRRK2 kinase activity, as well as antisense oligonucleotides to target the protein itself, are in clinical trials. Despite this, exactly how LRRK2 contributes to PD pathogenesis remains to be determined, and definitive biomarkers to track LRRK2 function are still required. Such biomarkers can be useful for monitoring the pharmacodynamic response of LRRK2 therapeutics and/or understanding the relationship between LRRK2 and the clinical progression of PD. Moreover, biomarkers that can identify increased LRRK2 levels or activity beyond just carriers of pathogenic LRRK2 mutations will be important for expanding LRRK2 therapeutics to other PD populations. This review summarizes recent findings regarding biomarkers of LRRK2.

## Introduction

Parkinson’s disease (PD) is an increasingly common neurodegenerative disorder that presents clinically with motor symptoms, including bradykinesia, resting tremor, rigidity and postural instability [1]. PD motor symptoms result from the selective degeneration of dopamine-producing neurons in the midbrain, a process which may begin decades prior to the manifestation of clinical symptoms [2]. Dopamine replacement therapies are the mainstay of symptomatic PD treatment; however, side effects and reduced efficacy over time are problematic [3]. Moreover, there is presently no way to slow or halt PD pathology from progressing. In addition to the degeneration of dopamine neurons, PD is also defined by the intraneuronal accumulation of proteinaceous inclusions, termed Lewy bodies, that are enriched in the protein alpha-synuclein [4]. Lewy pathology does not appear randomly but spreads through the brain in an organized or staged pattern over a time course of decades [5], resulting in widespread neuronal dysfunction. The spreading of Lewy pathology is thought to contribute to numerous non-motor PD symptoms, including hyposmia, gastro-intestinal dysfunction, sleep behavior difficulties, depression, anxiety and cognitive decline [6]. There are also no current ways of preventing the spread of Lewy pathology, thus effective therapies for the treatment of PD are clearly needed.

A potential therapeutic target for PD that has garnered much excitement is leucine-rich repeat kinase 2 (LRRK2). A serine/threonine protein kinase and member of the receptor-interacting protein kinase family, LRRK2, was confirmed as being genetically linked to PD in 2004 [7,8]. There are now more than 200 variants described for *LRRK2*, with at least 25 pathogenic for an increased risk of familial PD [9]. Polymorphisms in *LRRK2* have also been linked to sporadic PD [10], and collectively, it is thought that *LRRK2* variation may contribute up to 10% of familial PD and 5% of sporadic PD cases, although estimates vary substantially as LRRK2 mutations appear enriched in certain ethnic backgrounds [11]. Importantly, PD associated with missense mutations in LRRK2 appears largely clinically indistinguishable from sporadic PD [12], potentially suggesting a mechanistic overlap and that LRRK2 may also play a role in common sporadic PD. Although pathological differences do occur, with LRRK2 mutations having a propensity to give rise to tau pathology, which is usually more associated with Alzheimer’s disease, rather than classical alpha-synuclein Lewy pathology [13].

The strong genetic associations to PD were a driving factor in advancing LRRK2 therapeutics, and this was further facilitated by early findings that the most common pathogenic LRRK2 missense mutations increase the enzyme's catalytic protein kinase activity [14,15]. A large number of programs to develop LRRK2 kinase inhibitors ensued, identifying potent compounds with high selectivity and sensitivity (reviewed by Morez et al. [16]). The development of potent inhibitors of LRRK2 has been relatively rapid, advancing to a stage where lead compounds have passed phase 1 safety trials [17,18] and are now undergoing phase 2 testing in patient populations. Further development of inhibitors to investigate the therapeutic potential of type-2 inhibitors [19] and LRRK2 mutation-selective inhibitors [20,21] is ongoing. In addition to kinase inhibitors, other approaches are being developed to target LRRK2, including antisense oligonucleotides and proteolysis-targeting chimeras [22–24], all with the collective goal of reducing LRRK2 protein and/or its activity to determine whether this will ameliorate PD pathology.

Indeed, therapeutic development has outpaced understanding of the biological function of LRRK2, which is still not completely clear. LRRK2 has been implicated in lysosomal function, mitochondrial function, immunity and ciliogenesis. It remains to be determined exactly how LRRK2 contributes to PD pathology and the impact that LRRK2 therapeutics may have on these biological functions in a clinical setting. An incomplete understanding of LRRK2 function also affects the development of biomarkers that can be used as target engagement markers in clinical trials, or importantly, the identification of sporadic PD patients with LRRK2 dysfunction, who may be stratified into LRRK2 therapeutic trials. This review summarizes the current understanding of LRRK2 biomarkers, either using LRRK2 or its substrates directly as biomarkers, or downstream functional readouts dependent on LRRK2 activity. The development of LRRK2 biomarkers to aid in the design and interpretation of clinical trials is an important and active area of research.

### Direct measurement of LRRK2 as a biomarker

#### LRRK2 phosphorylation

The earliest methods of assessing LRRK2 activity in the absence of any known substrates or signaling pathways relied on autophosphorylation. Increased incorporation of radioactive ATP into mutant forms of LRRK2 was the first evidence that pathogenic PD missense mutations could increase LRRK2 activity [14]. More than 70 LRRK2 autophosphorylation sites have subsequently been mapped (for a comprehensive review on LRRK2 autophosphorylation sites, please refer to Marchand et al. [25]) and several antibodies generated, with assessment of the Ser1292 autophosphorylation site, in particular, confirming that PD pathogenic missense mutations increase the kinase activity of LRRK2 [26]. The measurement of LRRK2 Ser1292 phosphorylation has also been used to indicate increased LRRK2 activity in sporadic PD postmortem brain [27] and exosomes isolated from urine [28]. LRRK2 Ser1292 phosphorylation was also detectable in cerebrospinal fluid (CSF) and increased in PD patients with cognitive impairment [29]. However, in general, LRRK2 autophosphorylation is intrinsically low and difficult to detect without additional purification steps, hampering routine biomarker use in clinical studies. However, ongoing efforts to develop and optimize assays for measuring Ser1292 (e.g., [30]), or other autophosphorylation sites, may facilitate stratification biomarkers for identifying sporadic PD patients with increased LRRK2 activity.

**Figure 1:**
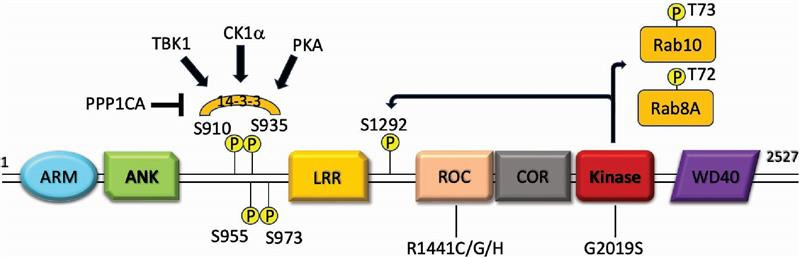
Leucine-rich repeat kinase 2 (LRRK2) phosphorylation. The domain structure of LRRK2 showing G2019S and R1441C/G/H pathogenic mutations, the Ser1292 autophosphorylation site, Rab substrate proteins, and Ser910 and Ser935 14-3-3 binding sites.

Instead of autophosphorylation, the LRRK2 Ser910 and Ser935 phosphorylation sites provide a more robustly detectable measurement of LRRK2 activity, at least in terms of developing assays for measuring LRRK2 inhibitor target engagement. The LRRK2 Ser910 and Ser935 phosphorylation sites are located N-terminal to the enzyme's leucine-rich repeats and are important for binding of LRRK2 to 14-3-3 proteins (Figure 1) [31]. These residues, along with the nearby Ser955 and Ser973, are not LRRK2 autophosphorylation sites but are constitutively phosphorylated by upstream kinases, including casein kinase 1-alpha [32], TANK-binding kinase 1 (TBK1) [33] and protein kinase A [34]. Thus, unlike autophosphorylation sites, increased phosphorylation of these residues does not necessarily imply increased kinase activity. However, numerous studies have robustly demonstrated reduced phosphorylation on all four of these residues, with the loss of LRRK2 kinase activity following treatment with ATP-competitive type-1 inhibitors. The exact mechanisms by which the loss of LRRK2 kinase activity leads to dephosphorylation of Ser910 and Ser935 remain to be determined but may involve stabilization of LRRK2 in an active conformation leading to exposure of the serine residues to protein phosphatase 1 (PPP1CA) [35]. Levels of LRRK2 Ser935 phosphorylation have been extensively studied as excellent antibody-based reagents have been developed for its measurement. LRRK2 and Ser935 phosphorylation are readily detectable in blood monocytes and neutrophils, and the treatment of blood cells with LRRK2 inhibitors *ex vivo* readily shows a dose-dependent reduction in Ser935 phosphorylation [36]. Moreover, LRRK2 Ser935 phosphorylation is significantly reduced in blood cells following the treatment of healthy participants with a LRRK2 inhibitor during phase-1 testing [17,18]. Thus, levels of LRRK2 Ser935 remain a valid biomarker of LRRK2 inhibitor target engagement, but there are caveats that should be noted. In particular, pathogenic LRRK2 mutations can also affect LRRK2 Ser910, Ser935, Ser955 and Ser973 phosphorylation. In a screen of 100 LRRK2 variants, several mutations reduced the levels of LRRK2 Ser935 phosphorylation, including the more commonly studied pathogenic kinase activating mutations R1441G/H, Y1699C, I2020T and G2385R [37]. Thus, careful genotyping of patients for LRRK2 mutations is required if employing LRRK2 Ser935 phosphorylation as a target engagement marker in clinical trials. Indeed, the use of LRRK2 Ser935 phosphorylation may be limited if trials seek to enrich for patients harboring pathogenic kinase-activating mutations. Although it should be noted that the most common pathogenic LRRK2-activating mutation, G2019S, does not reduce Ser935 phosphorylation [37], and further mechanistic understanding of how different LRRK2 variants affect the phosphorylation of these biomarker residues is still required. Secondly, reduced LRRK2 Ser910, Ser935, Ser955 and Ser973 phosphorylation following LRRK2 inhibitor treatment is only observed for ATP-competitive type-1 inhibitors, which trap LRRK2 in an active conformation. Type 1 inhibitors include the widely used MLi2 tool compound [38], as well as the DNL151/BIIB122 inhibitor employed in clinical trials [17]. However, the recent identification of type-2 inhibitors that target and trap LRRK2 in an inactive conformation shows that these compounds do not reduce phosphorylation of this serine cluster [39], and thus, the use of these phosphorylation sites is not an effective readout of type-2 inhibitor target engagement. Finally, as these are not autophosphorylation sites, phosphorylation on LRRK2 Ser910 and Ser935 is also subject to regulation by upstream kinases, which can also confound LRRK2 inhibitor-induced dephosphorylation. For example, treatment of immune cells with lipopolysaccharide (LPS) results in the activation of the upstream kinase TBK1, and levels of Ser935 phosphorylation remain stable even in the presence of LRRK2 inhibitors [33]. In summary, LRRK2 Ser935 phosphorylation has been successfully validated as a pharmacodynamic biomarker of LRRK2, but some considerations are needed when applying this biomarker in patient studies.

#### LRRK2 substrate phosphorylation

A fundamental breakthrough for the LRRK2 field came with the discovery of a subset of Rab GTPase proteins as substrates of LRRK2 [40]. The Rab GTPase family comprises ~60 members that catalyze the hydrolysis of GTP to GDP and form an integral component of intracellular membrane trafficking [41]. A comprehensive proteomic analysis identified Rab10 Thr73, a conserved residue located in the GTPase switch II domain, as a LRRK2 phosphorylation site [40]. *In vitro* analyses extended the number of endogenous Rab proteins phosphorylated by LRRK2 to at least 10, including Rab3a, Rab8a, Rab12, Rab35 and Rab43, which all contain the conserved equivalent of Rab10 Thr73 [42]. Although other substrates have been proposed for LRRK2, Rab GTPase proteins currently comprise the most robustly validated substrates across many research groups, with increasingly well-developed reagents for their study. In particular, highly sensitive antibodies against Thr73 Rab10 have facilitated research into this LRRK2 substrate [43]. Importantly, kinase-activating pathogenic mutations all increase phosphorylation of Thr73 Rab10 [37], and similar to the LRRK2 Ser935 phosphorylation site, Rab10 phosphorylation is reduced in a dose-dependent manner with LRRK2 kinase inhibitors in cell and animal models. Although the use of the mobility shift phos-tag approach to measure Rab10 phosphorylation does show that the stoichiometry of Rab phosphorylation by LRRK2 is low, at least under baseline conditions [44], and therefore sensitive detection methods are required.

Rab10 is also highly expressed in neutrophils and peripheral monocytes, precipitating studies to investigate Rab10 phosphorylation in clinical cohorts using blood samples. In regard to using Rab10 phosphorylation as a target engagement biomarker, *ex vivo* [45–47] and *in vivo* patient studies [17,18] have confirmed reduced Rab10 phosphorylation in blood mononuclear cells upon exposure to LRRK2 kinase inhibitors; thus, Rab10 phosphorylation can be used as a target engagement biomarker in clinical trials. Importantly, Rab10 phosphorylation is also reduced by type-2 kinase inhibitors [39], providing an advantage over the use of LRRK2 Ser935.

In addition to a target engagement biomarker, there is also much interest in whether Rab10 phosphorylation comprises a disease progression biomarker, or especially a stratification biomarker that may identify sporadic PD patients with high levels of LRRK2 activity, who may be enrolled into LRRK2-targeted clinical trials. An initial multi-site study employing immunoblotting suggested no difference in Rab10 phosphorylation in neutrophils from sporadic PD patients compared with controls, although there was a correlation between Rab10 phosphorylation and motor disease severity, as well as peripheral levels of several inflammatory cytokines [46]. Because Rab10 and LRRK2 are predominantly expressed in monocytes, flow cytometry has been employed to measure cell type-specific Rab10 phosphorylation in heterogeneous peripheral blood mononuclear cell (PBMC) samples, with initial studies again suggesting no difference in Rab10 phosphorylation between PD and control monocyte subsets [48]. However, Rab10 phosphorylation in neutrophils and monocytes is notably heterogeneous, and a lack of a significant group effect, therefore, does not preclude that there may be a subpopulation of idiopathic PD patients with elevated peripheral LRRK2 activity. Indeed, more advanced approaches are continuing to address this question. One approach is to challenge immune cells and potentially exacerbate minor differences seen at baseline. For example, challenge of immune cells with interferon gamma resulted in significantly increase Rab10 phosphorylation in PD patient monocytes compared with controls [48]. The use of more sensitive single molecule array technology has recently demonstrated the presence of phosphorylated Rab10 in serum [49]. In this study, Rab10 phosphorylation could be detected in ~75% of the cohort, with the remaining 25% below the lower limit of detection. Moreover, elevated Rab10 phosphorylation could be detected in a subset of idiopathic PD patients, and this was associated with worse motor symptoms and elevated expression of inflammatory genes [49], consistent with the outcomes of original immunoblotting studies. Additional approaches include the use of parallel reaction monitoring mass spectrometry to simultaneously assess a panel of Rab phosphorylation sites in immune cells [50], as well as expanding to other biofluids such as the detection of Rab phosphorylation in urinary extracellular vesicles [51,52]. Thus, there is some promise of Rab10 or other LRRK2 Rab substrates to act as stratification or disease progression biomarkers, but further efforts to translate this concept are still required. A greater understanding of the mechanisms by which LRRK2 regulates Rab phosphorylation is likely to be important for translational efforts. In particular, it is important to understand the circumstances under which LRRK2 regulates distinct Rab proteins in different cell types under different conditions, and to determine whether and how Rab proteins mechanistically contribute to PD.

#### LRRK2 levels

While monitoring phosphorylation of levels of LRRK2 and its substrates can inform on enzyme activity, there is also substantial interest in the levels of LRRK2 protein itself, especially for therapeutic strategies aimed at reducing LRRK2 protein levels. As outlined above, LRRK2 is highly expressed in peripheral immune cells, such as monocytes and neutrophils, making these cell subsets a useful peripheral source of LRRK2. LRRK2 can also be measured in whole blood and PBMC lysates using ELISA, but within-subject variability is much greater in PBMCs [47,53], likely due to the heterogeneity in cellular composition of PBMC samples. Several single-site cross-sectional studies using different methodologies have reported no difference in PBMC LRRK2 levels in peripheral immune cells between control and PD patients [46,53–55]. However, at least one study has reported increased levels of LRRK2 with PD in the less variable neutrophil population, whereas no difference was observed in PBMCs obtained at the same time from the same patient [46], suggesting additional steps to isolate distinct cell populations for increased sensitivity seem warranted. In the same study, there was no correlation of increased levels of LRRK2 with increased Rab10 phosphorylation, suggesting that LRRK2 levels themselves may not infer enzyme activity. Indeed, lower levels of LRRK2 protein have been measured in PBMCs [47] and brain tissue [56] from carriers of the kinase-activating LRRK2 G2019S mutation. Intriguingly, LRRK2 kinase inhibitors have also been suggested to reduce LRRK2 levels by promoting its proteasomal degradation [57], and reduced levels of LRRK2 protein were observed in chronically inhibitor-treated mice and non-human primates [58,59], albeit in a tissue-specific manner. Thus, although LRRK2 can be reliably detected in certain tissues, the measurement of total LRRK2 is unlikely to be a simple substitute for inferring LRRK2 kinase activity.

In addition to blood cells, LRRK2 can also be detected in CSF using sensitive mass spectrometry approaches [60], or enrichment of LRRK2-containing extracellular vesicles [61]. In a study of 106 patients, CSF levels of LRRK2 were significantly up-regulated in PD patients with the LRRK2 G2019S mutation, compared with controls, sporadic PD and non-manifesting LRRK2 mutation carrier groups [60]. Intriguingly, levels of LRRK2 were decreased in postmortem brain tissue from LRRK2 G2019S mutation carriers, in both the disease-affected frontal cortex and disease-unaffected occipital cortex [56], possibly explaining higher levels in CSF. However, whether levels of LRRK2 in CSF are truly representative of levels of LRRK2 in human brain remains unknown, as there are currently no methods to assess LRRK2 in living brain cells. Importantly, reduced levels of LRRK2 in CSF were observed in healthy patients dosed with LRRK2 inhibitors as part of phase 1 clinical trials, suggesting the utility of this approach to demonstrate central nervous system effects of LRRK2 inhibitors [17]. CSF levels of LRRK2 are also increased with age [60], and in a recent study, the highest levels of CSF LRRK2 were found in patients with dementia [29]. LRRK2 can also be measured in extracellular vesicles isolated from urine, a less invasive sample to collect. As for CSF, LRRK2 levels in isolated urinary exosomes have also been reported as elevated in LRRK2 G2019S mutation carriers, with no difference observed for sporadic PD compared with control [52]. However, elevated levels of LRRK2 in urine from LRRK2 G2019S carriers have not always been observed [62,63].

In summary, LRRK2 protein is readily detectable in peripheral blood samples using standard approaches, or the lower levels of LRRK2 present in CSF and urine can be detected with more advanced approaches. Monitoring levels of LRRK2 in these biosamples during clinical trials may inform on LRRK2 pharmacodynamics and/or be important for interpreting results from assays measuring LRRK2 or Rab phosphorylation. A relatively small number of studies to date suggest that LRRK2 levels are not sufficiently altered in sporadic PD to allow for patient stratification, and the direct measurement of LRRK2 in living brain cells is not currently possible; the extent to which peripheral measures of LRRK2 inform on central nervous system pathobiology still needs to be determined.

### LRRK2 functional biomarkers

While a comprehensive review on the proposed biological functions of LRRK2 is beyond the current scope, there are aspects of LRRK2 biology that currently stand out as potential functional biomarkers of LRRK2 activity.

#### LRRK2 and lysosomal function/BMP

A large body of evidence has confirmed a role for LRRK2 in regulating lysosomal function. LRRK2 can be activated in cellular models by lysomotropic agents, that is compounds that can penetrate and accumulate in lysosomes to exert a function, such as chloroquine and L-Leucyl-L-Leucine methyl ester hydrobromide (LLOMe). Broadly, lysomotropic agents that perturb lysosomal membranes or function can result in the recruitment of LRRK2 to the damaged lysosome, increasing LRRK2 activity and phosphorylation of downstream Rab substrates [64–69]. As a result, LRRK2 likely plays a role in lysosomal homeostasis, with several defective lysosomal phenotypes having been described in LRRK2 mutation cell and animal models (for a more detailed review, please refer to Madureira et al. [70]). Indeed, many of these phenotypes have been closely examined in regard to the safety of LRRK2 kinase inhibitors. In one such study employing non-human primates, the lysosomal phospholipid di-22:6 Bis(monoacylglycerol)phosphate (BMP) was assessed in urine, plasma and CSF following *in vivo* dosing with LRRK2 kinase inhibitors [58]. BMP has previously been proposed as a biomarker of excessive accumulation of phospholipids, which can occur with lysosomal storage disease or treatment with a class of lysomotropic agents collectively known as cationic amphiphilic drugs (CADs) [71]. Study authors noted that increased vacuolation of type-2 pneumocytes in the lungs of LRRK2 inhibitor-treated primates resembled phospholipidosis phenotypes reported for CAD treatment; hence, levels of the CAD-induced phospholipidosis biomarker di-22:6 BMP were assessed by mass spectrometry. Rather than the expected increase, however, urine levels of di-22:6 BMP were significantly reduced in LRRK2 kinase inhibitor-treated primates and also subsequently found decreased in urine from LRRK2 deficient rodents [58]. These findings facilitated additional studies in patient cohorts, which have consistently found elevated levels of di-22:6 BMP and other BMP isoforms in urine from LRRK2 mutation carriers, both manifesting and non-manifesting for PD [72–74]. One large cohort study found no correlation of urine BMP levels to clinical PD data and also no overall group change of urine BMP levels in sporadic PD patients compared with controls [72]. This suggests limited utility of urine BMP levels as a biomarker of PD progression, and further work to examine how BMP levels relate to other measures of LRRK2 kinase activity in sporadic PD patients is needed to determine any potential as a stratification biomarker for this population. Nonetheless, urine levels of di-22:6 BMP were decreased in a dose-dependent manner in healthy individuals during a phase 1 study of LRRK2 kinase inhibitors [17], thereby serving as a pharmacodynamic biomarker that may also inform on LRRK2-dependent lysosomal function.

#### LRRK2 and mitochondrial function

In Addition to lysosomal dysfunction, mitochondrial dysfunction has long been implicated in the pathogenesis of PD, with LRRK2 being associated with specific aspects of mitochondrial function. One such aspect is the regulation of mitochondrial quality control via the process of mitophagy. Mitochondrial quality control is very important in the context of PD as this prevents the accumulation of damaged mitochondria that can release reactive oxygen species that are detrimental to neurons. In fibroblasts from patients with the LRRK2 G2019S mutation, mitophagy is reported as both increased [75] and impaired [76,77]. In fibroblasts with LRRK2 mutations and impaired mitophagy, this defect was corrected by both knockout and kinase inhibition of LRRK2 [76,77]. This was later extended to mouse models, where basal mitophagy defects were measured in Lrrk2 R1441G [78] and Lrrk2 G2019S knockin mice [79], with defects in the G2109S mice being corrected with LRRK2 kinase inhibition *in vivo* [79]. However, in cells from the Lrrk2 R1441G mice [80] and in iPSC-derived dopamine neurons with the LRRK2 R1441C mutation [81], impaired mitophagy defects were not corrected with LRRK2 kinase inhibitors, potentially indicating complex cell type-dependent mechanisms. Resolving more of the pathway by which LRRK2 modulates mitophagy may uncover biomarker opportunities. However, a more translationally advanced LRRK2 mitochondrial biomarker may be the assessment of mitochondrial DNA damage. Mitochondria are unique organelles that contain their own 16 kb circular double-stranded DNA, which encodes for a number of genes that regulate mitochondrial function, such as respiratory chain subunits [82]. Mitochondrial DNA (mtDNA) is maternally inherited and can exist in various copy numbers. Due to close proximity to the reactive oxygen species producing respiratory chain complex and a limited localized DNA repair system, mtDNA is prone to developing mutations, and this underlies a number of mitochondrial diseases [82]. One of the earlier discoveries in the LRRK2 field was that neurons differentiated from iPSC containing LRRK2 G2019S or R1441C mutations had higher levels of mitochondrial DNA damage [83]. In the present study, a PCR-based assay was performed to allow relative quantification of mtDNA damage, using the assumption that under identical amplification conditions, the more mutations mtDNA contains will result in the accumulation of less PCR product [84]. Subsequent studies then demonstrated that mtDNA damage was elevated in immortalized lymphoblastoid cells from PD patients with LRRK2 G2019S mutation, and that this increase in mtDNA damage is reversible with inhibition of LRRK2 kinase activity [85,86]. Moreover, mtDNA damage levels appeared to be a more sensitive biomarker of LRRK2 activity than Rab10 phosphorylation, which was not significantly changed in the same LRRK2 G2019S lymphoblastoid cells [86]. Further improvements to improve quantification and throughput have been made, and importantly, the assay has also been extended to detecting mtDNA in PBMCs from PD patients [87]. In the present study, mtDNA damage was significantly increased in PBMCs from both PD patients and non-manifesting carriers of the LRRK2 G2019S mutation. Interestingly, mtDNA damage was also significantly up-regulated in sporadic PD patients without LRRK2 mutations [87]. This suggests potential utility as a stratification biomarker for identifying sporadic PD patients with increased LRRK2 activity, but it remains to be determined if increased mtDNA damage in sporadic PBMCs is indeed LRRK2 dependent. It was also noted that quantified mtDNA damage did not correlate with demographic or clinical PD data [87], suggesting that mtDNA damage may not comprise a disease progression biomarker. Finally, in the same study, a number of preanalytical variables were also assessed for their impact on the quantification of mtDNA damage, with results indicating an impact of cryopreservation/storage time/storage conditions to increase mtDNA damage. Best results were obtained by extracting mitochondrial DNA from fresh PBMC samples, which may not always be possible in large multi-site clinical trials. Nonetheless, PCR-based assessment of mtDNA damage remains a promising biomarker for LRRK2 pharmacodynamics and potentially the identification and stratification of PD patients who have elevated LRRK2 activity but without a LRRK2 mutation.

#### LRRK2 and centrosomal cohesion

Both lysosomal and mitochondrial dysfunction are heavily implicated in PD; however, further proteomic analysis of the LRRK2-Rab signaling pathway has also uncovered novel functions of LRRK2 with potential PD implications and biomarker potential. Soon after the discovery of Rab proteins as LRRK2 substrates, it was found that LRRK2-mediated phosphorylation of Rab8A and Rab10 increased interaction with the Rab-interacting lysosomal protein like 1 (RILPL1) and RILPL2 proteins [42]. The LRRK2 kinase activity-dependent interaction between Rab10 and RILPL1, in particular, has provided insight into novel roles of LRRK2 as a regulator of ciliogenesis [42,88,89] and centrosomal cohesion [90–92], with the latter being further developed as a PD biomarker. Within cells, RILPL1 localizes to the mother centriole within the centrosome, an organelle important for maintaining cellular structure and regulating cell division [93]. An important part of the cell division process is duplication of the centrosomes to allow formation of the mitotic spindle and segregation of chromosomes to produce two genetically identical cells. A close inspection of centrosome positioning in cells with pathogenic LRRK2 mutations showed a significantly larger percentage of cells harbored a split centrosome phenotype, where centrosomes were a distance of greater than 1.5 um apart [92]. This was suggestive of a premature centrosome splitting phenotype, which was readily reversible upon treatment with LRRK2 kinase inhibitors [92]. Further investigation demonstrated that the centrosomal cohesion phenotype was dependent on LRRK2-mediated phosphorylation of Rab8A and Rab10, and subsequent interaction with RILPL1 [90,92]. An increased number of cells showing centrosome splitting was also observed in immortalized lymphoblastoid cells from LRRK2 G2019S mutation carriers. In the present study, the centrosome splitting phenotype could be readily detected in all mutation lines studied and was a more sensitive readout of LRRK2 activity than immunoblot assessment of Rab10 phosphorylation [94]. The same phenotype has subsequently been observed in lymphoblastoid cells from carriers of the LRRK2 R1441G mutation, as well as non-manifesting carriers of both LRRK2 G2019S and R1441G mutations, and importantly in a subset of sporadic PD patients without LRRK2 mutations [95]. Moreover, even though the LRRK2-dependent cohesion splitting phenotype is easier to assess in actively dividing lymphoblastoid cells, it has also been observed in lymphocytes from biobanked PBMCs, both in LRRK2 mutation carriers and in a subset of sporadic PD patients [95]. This raises the possibility of using the centrosomal splitting phenotype as a stratification biomarker for identifying sporadic PD patients with increased LRRK2 activity. Higher throughput and potentially additional quantification measures to determine a stratification threshold for elevated LRRK2 activity would be beneficial, as well as expanding sample sizes and multi-site validation. Interestingly, although sample sizes are limited, there appears to be no correlation of centrosomal cohesion phenotype to PD clinical symptom scales, and whether this phenotype contributes mechanistically to PD pathogenesis remains to be determined. Due to largely overlapping signaling pathways, centrosomal cohesion phenotypes in the periphery could possibly constitute a biomarker of ciliogenesis in the brain, which has been more directly linked to degeneration of dopaminergic neurons [96–98]. Thus, although somewhat of a specialized assay, the measurement of centrosome splitting comprises a functional readout of LRRK2 kinase activity that is modulated by pathogenic mutations and LRRK2 kinase inhibitors.

### Summary and future directions

**Figure 2:**
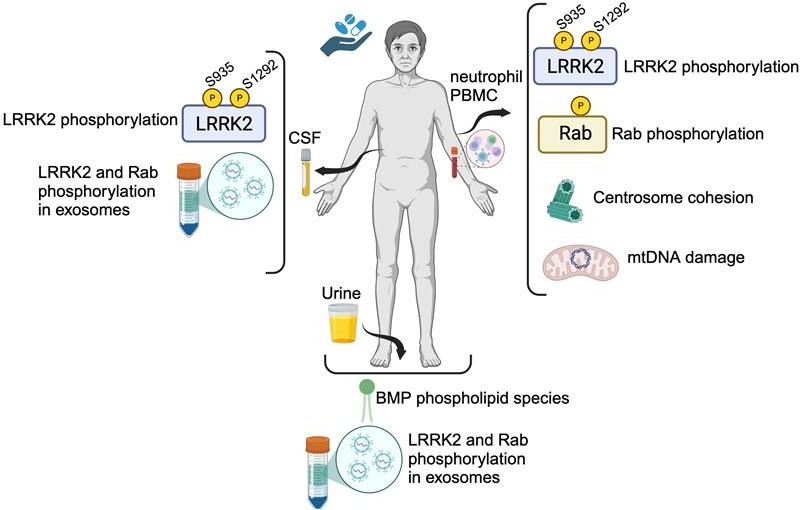
Current LRRK2 biomarkers for Parkinson’s disease. Biomarkers that can track the pharmacodynamic response to LRRK2 inhibitors have been developed for different biofluids, including blood immune cells, urine and CSF. Created in BioRender. Dzamko (2025) https://BioRender.com/t31u497

LRRK2 is a promising therapeutic target for the treatments of PD, for which drugs have been developed and are being tested in clinical trials. To facilitate clinical trials, biomarkers have been developed that can track the pharmacodynamic response for LRRK2 inhibitors (Figure 2). However, this likely still represents an early stage in LRRK2 biomarker development. Further advances in assay sensitivity to overcome the low and restricted expression of LRRK2 and low stoichiometry of substrate phosphorylation, coupled with an advanced understanding of the biological pathways that regulate LRRK2 activity, will be beneficial. In particular, defining the extent of cell type-dependent effects of LRRK2 and downstream Rab substrates, as well as developing tools for assessing additional LRRK2 Rab substrates, seems warranted.

The further development of LRRK2 stratification biomarkers to enable the potential treatment of sporadic PD patients with LRRK2 therapeutics is also required. Most studies assessing LRRK2 biomarkers in sporadic PD patients are still small, single-site cross-sectional studies, with validation in larger cohorts still required. Studies to assess multiple LRRK2 biomarkers in the same patient groups would also be beneficial for understanding the complex relationship between LRRK2, Rab phosphorylation and downstream functional readouts. It is also noteworthy that the majority of LRRK2 biomarkers are based on readouts from peripheral immune cells, and the extent to which LRRK2 in these cells may contribute to PD pathogenesis remains to be determined, as most peripheral LRRK2 readouts do not seem to associate with clinical disease rating scales. At present, however, there are no methods available to assess LRRK2 in living human brain, and the relationship between LRRK2 in CSF and brain tissue remains to be fully defined. As understanding of the biological pathways regulated by LRRK2 continues to advance, there will undoubtedly be additional functional readouts of LRRK2 and its enzyme activity that can be clinically translated.

## Abbreviations

BMP: Bis(monoacylglycerol)phosphate
CADs: cationic amphiphilic drugs
LRRK2: leucine-rich repeat kinase 2
PD: Parkinson’s disease
RILPL1: Rab-interacting lysosomal protein like 1
TBK1: TANK-binding kinase 1
iPSC: induced pluripotent stem cell
mtDNA: mitochondrial DNA
